# Mitragynine inhibits hippocampus neuroplasticity and its molecular mechanism

**DOI:** 10.1007/s43440-023-00541-w

**Published:** 2023-11-04

**Authors:** Suleiman Yunusa, Zurina Hassan, Christian P. Müller

**Affiliations:** 1 Centre for Drug Research, Universiti Sains Malaysia, 11800 Penang, Malaysia; 2 Department of Pharmacology, Bauchi State University Gadau, PMB 65 Itas/Gadau, Bauchi, Bauchi State Nigeria; 3 Department of Psychiatry and Psychotherapy, University Clinic, Friedrich-Alexander-University Erlangen-Nuremberg, Schwabachanlage 6, 91054 Erlangen, Germany; 4 Institute of Psychopharmacology, Central Institute of Mental Health, Faculty of Medicine Mannheim, University of Heidelberg, Heidelberg, Germany; 5 Psychiatric and Psychotherapeutic University Hospital, Friedrich-Alexander-University Erlangen-Nuremberg, Schwabachanlage 6, 91054 Erlangen, Germany

**Keywords:** Mitragynine, Cognitive deficit, Hippocampal synaptic transmission, fEPSP, Western blot

## Abstract

**Background:**

Mitragynine (MIT), the primary indole alkaloid of kratom (*Mitragyna speciosa*), has been associated with addictive and cognitive decline potentials. In acute studies, MIT decreases spatial memory and inhibits hippocampal synaptic transmission in long-term potentiation (LTP). This study investigated the impacts of 14-day MIT treatment on hippocampus synaptic transmission and its possible underlying mechanisms.

**Methods:**

Under urethane anesthesia, field excitatory post-synaptic potentials (fEPSP) of the hippocampal CA1 region were recorded in the Sprague Dawley (SD) rats that received MIT (1, 5, and 10 mg/kg), morphine (MOR) 5 mg/kg, or vehicle (*ip*). The effects of the treatments on basal synaptic transmission, paired-pulse facilitation (PPF), and LTP were assessed in the CA1 region. Analysis of the brain's protein expression linked to neuroplasticity was then performed using a western blot.

**Results:**

The baseline synaptic transmission's amplitude was drastically decreased by MIT at 5 and 10 mg/kg doses, although the PPF ratio before TBS remained unchanged, the PPF ratio after TBS was significantly reduced by MIT (10 mg/kg). Strong and persistent inhibition of LTP was generated in the CA1 region by MIT (5 and 10 mg/kg) doses; this effect was not seen in MIT (1 mg/kg) treated rats. In contrast to MIT (1 mg/kg), MIT (5 and 10 mg/kg) significantly raised the extracellular glutamate levels. After exposure to MIT, GluR-1 receptor expression remained unaltered. However, NMDAε2 receptor expression was markedly downregulated. The expression of pCaMKII, pERK, pCREB, BDNF, synaptophysin, PSD-95, Delta fosB, and CDK-5 was significantly downregulated in response to MIT (5 and 10 mg/kg) exposure, while MOR (5 mg/kg) significantly raised synaptophysin and Delta fosB expression.

**Conclusion:**

Findings from this work reveal that a smaller dose of MIT (1 mg/kg) poses no risk to hippocampal synaptic transmission. Alteration in neuroplasticity-associated proteins may be a molecular mechanism for MIT (5 and 10 mg/kg)-induced LTP disruption and cognitive impairments. Data from this work posit that MIT acted differently from MOR on neuroplasticity and its underlying mechanisms.

**Graphical abstract:**

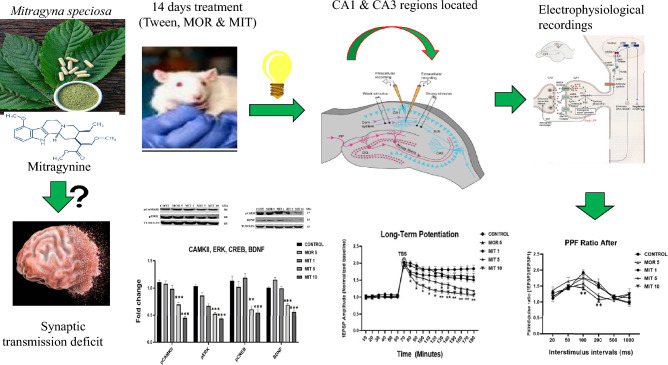

**Supplementary Information:**

The online version contains supplementary material available at 10.1007/s43440-023-00541-w.

## Introduction

Mitragynine (9-methoxy-corynantheidine) (MIT) is the most studied active indole alkaloid from kratom (*Mitragyna speciosa*) that constitutes more than 60 percent of the total alkaloids in kratom [1, 2]. Kratom and MIT's potential for addiction and cognitive deficits appear to be debatable in light of human studies [3], and they may even be used therapeutically to treat other drug addictions [4–6]. However, long-term use can impair cognitive functions. Animal studies revealed learning and memory deficits after either short or prolonged exposure to kratom or MIT [7–12].

The investigational mechanism in rodent CA1 hippocampal slices revealed that acute and sub-chronic treatments with standardized kratom extracts could significantly decrease non-potentiated field excitatory post-synaptic potentials (fEPSPs) and block long-term potentiation (LTP) [13, 14]. Data from our lab also showed that an acute MIT treatment reduced synaptic transmission and impaired spatial learning in the Morris water maze by significantly reducing fEPSP amplitude during LTP in the CA1 region of the hippocampus of urethane-anesthetized rats [15].

It is believed that the brain modifies the strength of synaptic connections between neurons to encode and store memory. Chronic use of substances with abuse potential alters synaptic plasticity in learning-associated brain circuits gradually and cumulatively, which leads to long-term behavioral changes [16–18]. The mechanism of synaptic plasticity in the hippocampus posits that a high-frequency stimulation at the CA3 pre-synaptic pyramidal neuron allows the entry of Ca^2+^ into the CA1 post-synaptic pyramidal neurons as a result of N-methyl-D-aspartate receptor (NMDA-R) activation [19–21], which modulates alpha Ca2 + /calmodulin-dependent protein kinase II (CaMKIIα). At the Schaffer collateral–commissural pathway of the adult rat's hippocampus, CaMKIIα is abundant and is necessary for LTP-dependent protein activation (18), which is not only core to the synaptic plasticity, learning, and memory [22] but also potential for drug addiction development [23]. CaMKIIα becomes auto-phosphorylated and mediates some intracellular cascades that can result in a long-lasting increase in synaptic transmission [24]. The latter is accomplished by phosphorylating specific target proteins and transcription factors, such as cyclic AMP-response element binding protein (CREB), which plays a role in forming long-term memory [25]. CREB phosphorylation, particularly at serine 133, activates brain-derived neurotrophic factor (BDNF), leading to neuronal outgrowth, and hence the strengthening of synaptic transmission [26]. Neuronal survival, morphogenesis, LTP, and synaptic plasticity in the hippocampus are greatly influenced by BDNF [27–29]. The protein kinases known as extracellular signal-regulated kinases (ERKs) are highly present in the central nervous system and significantly contribute to neuroplasticity [27]. Activated ERK (pERK) directly phosphorylates CREB, and therefore remains a critical protein during LTP induction [27, 30].

The synaptic vesicle membrane protein, synaptophysin is associated with both axonal growth and brain plasticity and is still a crucial pre-synaptic molecule for the development and maintenance of synapses [31–33]. The post-synaptic density protein 95 (PSD-95) controls the actions of glutamate receptors, including α-amino-3-hydroxy-5-methyl-4-isoxazole propionic acid receptors (AMPA-R) and NMDA-R, which are essential for hippocampal neuroplasticity [34]. Activity-dependent neuroplasticity pathways underpinning learning and memory formation have been linked to synaptophysin and PSD-95 [35]. Delta fosB expression also depends on Ca^2+^ influx and ERK activation [36]. Cyclin-dependent protein kinase 5 (CDK-5) is a synaptic plasticity biomarker since it promotes neuronal connection and NMDA-R conductance in the hippocampus [37].

The mechanisms underlying MIT-induced synaptic plasticity deficits in the hippocampal CA1 region remains unresolved. Thus, this study aimed to examine the impact of sub-chronic MIT administration on synaptic transmission and its neural mechanisms in the hippocampus of rats. The selection of MIT doses (1, 5, and 10 mg/kg) in this study was based on the earlier reported data, as the highest dose tested (10 mg/kg) was found to impair spatial memory [15] and disrupted LTP in rats that received urethane anaesthesia [15, 38]. Morphine (MOR) 5 mg/kg was chosen as a reference drug for this study because of its ability to affect cognitive function in rodents [10, 13]. In addition, MIT acts partially on the opioid receptor in a similar way to MOR [39]. The selection of 5 mg/kg dose for MOR in this study was based on our previous study [15].

## Experimental procedures

### Animals

All experimental procedures received approval from the Institutional Animal Care and Use Committee (IACUC), Universiti Sains Malaysia {USM/IACUC/2020/(124)(1082)}. The research was carried out in accordance with the bioethical law applicable in the EU/USA. Forty (40) male Sprague Dawley (SD) rats weighing 200–300 g were obtained from the Animal Research and Service Centre (ARASC) at Universiti Sains Malaysia. The rats were housed in cages of not more than five rats per cage and were provided with unlimited access to water and food, and a 12-h light and dark cycle was adhered to (07:00–19:00) daily. Before any experiment, all animals were acclimated to a temperature and humidity-controlled environment for at least 7 days.

### Drugs

MIT used in this study was extracted in-house from the kratom leaves using the procedure outlined by Ref. [38]. High-performance liquid chromatography verified the MIT's 98% purity (HPLC). MOR hydrochloride (Lipomed Pharm., Batch: 35.IB0.2) and MIT solutions were freshly prepared daily using 20% Tween 80 (vehicle) prior to intra-peritoneal (*ip*) administration.

### In vivo electrophysiology

Animals were treated (*ip*) with control vehicle (20% tween 80), MOR (5 mg/kg), and MIT (1, 5, and 10 mg/kg) for 14 days and used in electrophysiological studies as described previously [15]. Urethane at a dose of 2 g/kg was given to the animals in four separate doses at a rate of 0.5 g/kg each after every 20 min. Urethane was selected for this study because it can induce anesthesia without causing any interference with neurotransmission in a range of subcortical regions and the peripheral nervous system [39]. In addition, urethane is known to produce long-term stability of physiological signals in rats without markedly affecting both cardiovascular and respiratory systems [40].

Animals were mounted on a stereotaxic frame for surgery. Subcutaneously, a local analgesic agent (Xylocaine, 5 mg/kg) was injected at the incision site. The animals' body temperatures were kept at 36–37 °C throughout the trial using a blanket and an electric heating pad.

The CA1 and CA3 regions were located by taking coordinate readings from the bregma and the surface of the skull. The coordinate of CA1 (AP: − 4.2 mm, ML: -3.0 mm; V: 3.0 mm) and CA3 (AP: − 4.2 mm; ML: + 3.0 mm: V: − 4.0 mm. A stimulating electrode (SNE 100, MicroProbes, USA) was implanted at the CA3 area to stimulate the Schaffer collateral/commissural route (0.2 ms at 0.05 Hz). A recording electrode (Insulated Steel Wire, A-M Systems, USA) was implanted at the CA1. Two screws were drilled into the frontal cortex as the recording electrode's ground and reference. Final ventral adjustments were made to the CA3 stimulating and CA1 recording electrodes to reach the maximal fEPSP amplitude.

The CA3 region was stimulated with increasing intensity from 0.1 to 1.0 mA to create an input/output (I/O) curve. The intensity that produced an fEPSP amplitude of approximately 50–60% of the maximum in CA1 was utilized for the subsequent measurement of paired-pulse facilitation (PPF) and LTP. PPF was measured by delivering pairs of stimulation pulses (0.2 ms duration) with interstimulus intervals (ISIs) of 20, 50, 100, 200, 500, and 1000 ms to evaluate short-term plasticity.

Baseline fEPSPs were recorded for 60 min to establish a steady baseline for the LTP. LTP was compared to the baseline fEPSP recordings by delivering one theta-burst stimulation (TBS) at the CA3 area immediately following the 60-min baseline recording. In a TBS train, ten stimulus bursts with five pulses at 100 Hz each were administered at a frequency of 5 Hz. After TBS was distributed, fEPSP recordings continued for the next 2 h. The electrophysiological signals were amplified and digitized using a PowerLab/4SP system (ADInstruments, Australia) at a rate of 10 kHz and stored in a PC. Offline analysis was performed using LabChart v. 7 software (ADInstruments). The treatment regimen was blinded to the experimenter who performed the electrophysiological recordings.

### Glutamate measurement

This study was conducted as outlined by Ref. [41]. Sodium pentobarbital (Dorminal 20% ®; Batch No 1609260–07, Alfasan, Holand), 100 mg/kg was used to euthanize the animals after the LTP experiment. The animal was decapitated before the structure isolation. The hippocampi (HP) and midbrain (MB) were isolated and immediately transported to a − 80 °C freezer for storage until needed. HP and MB were homogenized with an equal volume of chilled tissue protein extraction reagent (T-PER™, REF:78,510, LOT: WE320059; Thermo-scientific, USA) containing 1X protease inhibitor (Roche Diagnostics). The homogenates were spun for 10 min at 4 °C and 1000 g/min. The resulting supernatant was used for a colorimetric assay to determine the glutamate concentration. The glutamate level was quantified using a commercial kit (Elabscience^(R)^ Co. Ltd., USA) as specified by the manufacturer. The glutamate concentration was determined by reading the plates using a Bio-Rad microplate reader (Hercules, CA, USA) at 450 nm. The concentrations were extrapolated from standard curves and expressed in μmol/L.

### Assessment of protein expression with western blot

The glutamate colorimetric assay used HP supernatant for protein quantification and optimization. Total proteins in each sample were quantified using the Lowry microplate reader protocol as previously described [42] and subsequently used for western blot using standard SDS-PAGE Laemmli buffer system protocol [43] as previously conducted [44–46]. After determining the proper protein concentration, 50 µg of the proteins were added to the sample buffers (X4 LDS and 1 M DTT) and heated at 90 °C for 10 min before loading to the SDS–polyacrylamide gels. Electrophoresis began at 130 V for 90 min or until the stain reached the bottom of the gel. Separated proteins from the gels were transferred to nitrocellulose membranes (Bio-Rad) using a wet transfer cell for 45 min at 90 V. Membranes were then incubated in 5% (w/v) defatted dried milk to block unspecific binding during detection. The membranes were washed in TBS-T and subjected to immunodetection with primary antibodies incubated at 4 °C overnight on an orbital shaker. The membranes were then washed with TBS-T and re-incubated for 90 min in a secondary antibody HRP conjugate specific to the initial primary antibodies (Table 1). The membranes were then washed and re-incubated in streptavidin-HRP (1:3000 dilution) for 60 min. Membranes were again washed and subjected to colorimetric detection using Opti-4CN substrate (Bio-Rad, Batch No: 64450147, Expiry: 08–2023, USA). The substrate was prepared according to the manufacturer's specification, and the membrane was incubated (light off) in the substrate using an orbital shaker for 30 min or until clear protein bands were seen. The membranes were washed with deionized water for 15 min and protein abundance/expression was measured using ImageJ (NIH software). The densitometry values of each blot were normalized to tubulin levels and expressed as a ratio relative to the control group. Protein samples of each group were not pooled and acted as individual samples.

**Table 1:** List of the antibodies used for analysis

Primary antibody	Dilution	Source	Secondary antibody	Dilution	Source
GLUR-1 (A-6) (sc13152)	1:1000	Santa Cruz Biotechnology (USA)	Anti-mouse (SA0000I-1)	1:3000	Proteintech (USA)
NMDARε2 (A-8) (sc-13152)	1:1000	Santa Cruz Biotechnology (USA)	Anti-mouse (SA0000I-1)	1:3000	Proteintech (USA)
pCAMKIIΑ (A-1) (sc-13141)	1:1000	Santa Cruz Biotechnology (USA)	Anti-mouse (SA0000I-1)	1:3000	Proteintech (USA)
pERK (1/2) (sc-136521)	1:1000	Santa Cruz Biotechnology (USA)	Anti-mouse (SA0000I-1)	1:3000	Proteintech (USA)
pCREB-1 (Ser 133)	1:1000	Santa Cruz Biotechnology (USA)	Anti-mouse (SA0000I-1)	1:3000	Proteintech (USA)
BDNF (I11115)	1:1000	Santa Cruz Biotechnology (USA)	Anti-mouse (SA0000I-1)	1:3000	Proteintech (USA)
Synaptophysin (4329S)	1:1000	Cell Signalling (USA	Anti-rabbit (7074S)	1:3000	Cell Signalling (USA
PSD-95 (2507S)	1:1000	Cell Signalling (USA	Anti-rabbit (7074S)	1:3000	Cell Signalling (USA
Delta fosB (D3S8R)	1:1000	Cell Signalling (USA	Anti-rabbit (7074S)	1:3000	Cell Signalling (USA
CDK-5 (D1F7M)	1:1000	Cell Signalling (USA	Anti-rabbit (7074S)	1:3000	Cell Signalling (USA
αTubulin (66,031–1)	1:1000	Proteintech (USA)	Anti-mouse (SA0000I-1)	1:3000	Proteintech (USA)

### Statistical analysis

The normality of data distribution was checked using the Shapiro–Wilk normality test. Two-way ANOVA for repeated measures followed by Dunnett's post hoc test was used to analyze I/O, PPF, and LTP data. One-way ANOVA followed by Dunnett’s post hoc test was employed to analyze data from average fEPSP of the LTP last 60 min, colorimetric assay, and western blot. All statistical analyses were performed using GraphPad Prism (V. 9.0; GraphPad Software, Inc., San Diego, California USA) and a *p* value of < 0.05 was considered statistically significant for all studies.

## Results

### MIT effects on hippocampal I/O, PPF, and LTP

**Fig. 1:**
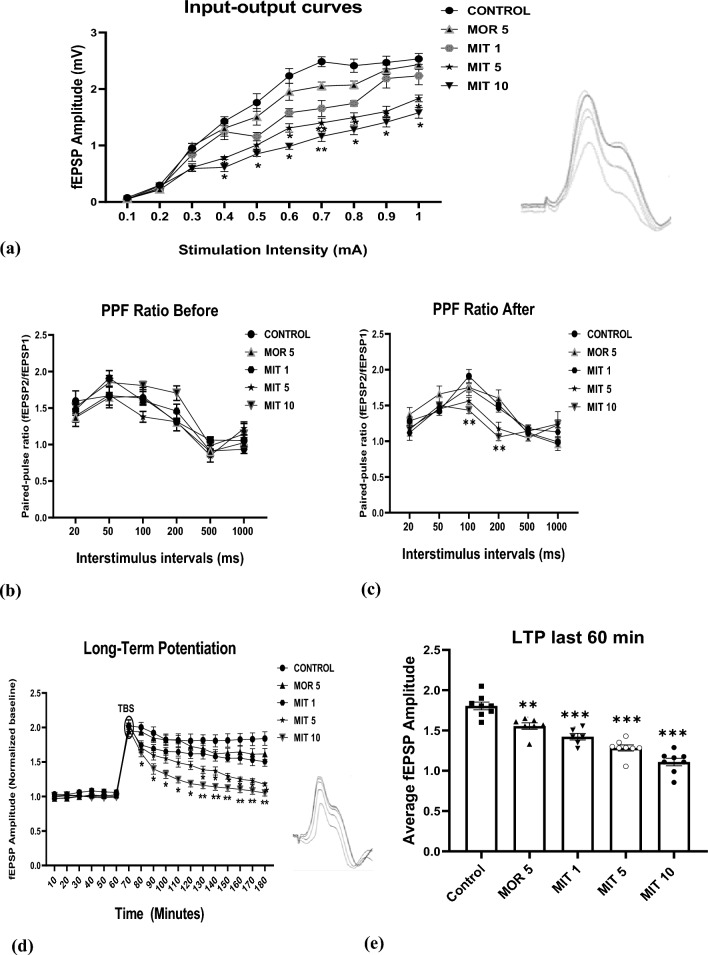
The effects of repeated administration of MOR (5 mg/kg) and MIT (1, 5, and 10 mg/kg) on field excitatory post-synaptic potentials (fEPSPs) in the hippocampal CA1 region of anaesthetized rats following (**a**) input–output curves generated with increasing intensities (0.1 to 1.0 mA); (**b** and **c**) PPF ratio measured before and after TBS at CA1 region following delivery of paired-pulse stimulation to CA3 at short interstimulus intervals of 20, 50, 100, 200, 500, and 1000 ms and calculating the amplitude of the two fEPSP; **d** LTP was assessed by recording a stable fEPSP amplitude for 60 min, followed by TBS delivery at the contralateral CA3 region and measurement of fEPSP continued for another 2 h and **e** average fEPSP amplitudes during the last 60 min of LTP. Data are presented as mean ± SEM and analyzed using two-way repeated measures ANOVA (**a**–**d**) and one-way ANOVA followed by the Dunnett’s post hoc test (**e**) (n = 8 animals/group).; * p < 0.05, ** p < 0.01, **** p < 0.0001 compared to control,. *MOR* morphine, *MIT* mitragynine, *CA1* cornu ammonis I, *CA3* cornu ammonis 3

MIT (5 and 10 mg/kg) significantly decreased the fEPSP amplitude during I/O curves generation from 0.4 to 1.0 mA intensities compared to the control group. MIT (1 mg/kg) and MOR (5 mg/kg) produced no significant reduction of the fEPSP amplitude throughout the experiment (Fig. 1a). A two-way ANOVA revealed significant effects of stimulation intensity (*F*_9350_ = *322, p* < 0.0001) and drug treatment (*F*_4350_ = 128.9*, p* < 0.0001), as well as of the interaction (*F*_36, 350_ = 5.171*, p* < 0.0001). Significant effects of the interstimulus interval (*F*_5206_ = 52.4*, p* < 0.0001) and of the drug treatment (*F*_4206_ = 3.986*, p* = 0.0039), but no significant interaction (*F*_20,206_ = 1.285*, p* = 0.1915) before the TBS were detected (Fig. 1b). A significant effect of interstimulus interval (*F*_*5199*_ = *41.50, p* < *0.0001*), drug treatment (*F*_4199_ = 4.078*, p* = 0.0034), and interaction (*F*_20,199_ = 3.431*, p* = 0.0034) after TBS was also revealed (Fig. 1c).

In the LTP study, a two-way ANOVA showed a significant effect of time (F_17,630_ = 158.6, *p* < 0.0001) and drug treatment (*F*_4630_ = 142.3, *p* < 0.0001), as well as of the interaction (F_68, 630_ = 5.332, *p* < 0.0001). MIT (5 mg/kg) significantly decreased the fEPSP amplitude, starting at 80 min and lasting until the end of the experiment (180 min). In contrast, MIT (10 mg/kg) significantly decreased the fEPSP amplitude starting at 130 min and lasting until the end of the experiment. Neither MIT (1 mg/kg) nor MOR 5 mg/kg altered LTP compared to the control throughout the testing period (Fig. 1d). In addition, a one-way ANOVA revealed that MIT (1, 5, and 10 mg/kg) significantly (*p* < 0.0001) decreased the average fEPSP amplitude during the LTP last 60 min compared to the control treatment. MOR (5 mg/kg) treatment yielded a significant difference (*p* < 0.0011) from the control group (Fig. 1e).

### MIT enhances glutamate levels in the hippocampus and midbrain

**Fig. 2:**
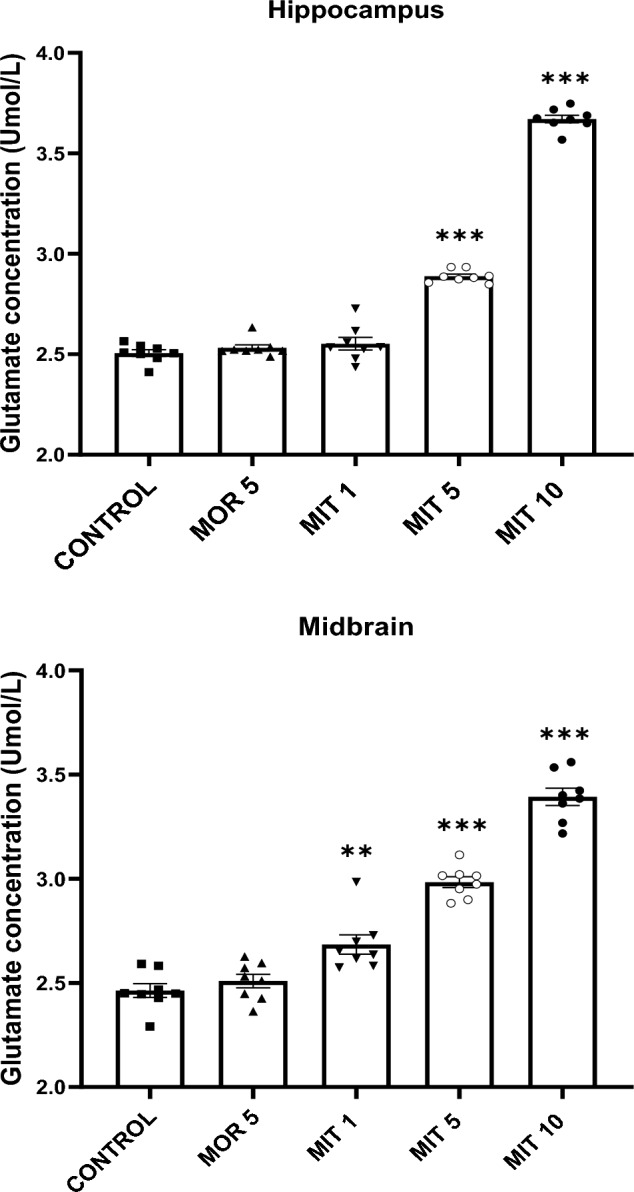
The effects of repeated administration of MOR (5 mg/kg) and MIT (1, 5, and 10 mg/kg) on glutamate concentration in the rats' hippocampus and midbrain using the colorimetric assay kit. Data are expressed as mean ± SEM and analyzed using one-way ANOVA followed by the Dunnett’s post hoc test (*n* = 8 replicates/group. **p < 0.001, ****p < 0.0001 compared to control. MOR—morphine, *MIT* mitragynine

A one-way ANOVA revealed a significant increase in the glutamate concentration in the hippocampus of MIT (5 and 10 mg/kg)-treated rats compared to the control (*F*_4,35_ = 624.4, *p* < 0.0001). MIT (1 mg/kg) and MOR (5 mg/kg) produced no significant difference in glutamate level compared to the control (*p* > 0.05). A significant and dose-dependent increase in glutamate concentration in the midbrain of all MIT-tested doses (1, 5, and 10 mg/kg) was observed as compared to control-treated rats (*F*_4,35_ = 110.8, *p* < 0.0001). MOR (5 mg/kg) did not change glutamate level in the midbrain compared to the control (*p* = 0.7913; Fig. 2).

### MIT reduces NMDAε2, but not GluR-1 expression

**Fig. 3:**
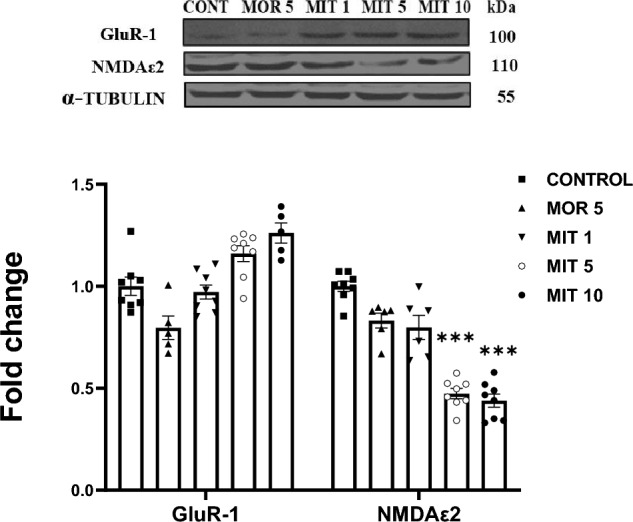
The effects of repeated administration of MOR (5 mg/kg) and MIT (1, 5, and 10 mg/kg)on GluR-1 and NMDAε2 expression in the rats' hippocampus using western blot. Data are expressed as mean ± SEM and analyzed using one-way ANOVA followed by the Dunnett’s post hoc test (*n* = 8 replicates/group). ***p < 0.0001. Values in each group were calculated in relation to the control group and alpha-tubulin served as a loading control. *MOR* morphine, *MIT* mitragynine, *NMDAε2* NMDA epsilon 2, *GluR-1* glutamate receptor 1

A one-way ANOVA and single-group comparisons did not show significant changes in GluR-1 in all the treatment groups compared to the control (MOR 5: *p* = 0.2705; MIT 1: *p* > 0.999; MIT 5: *p* = 0.2674; MIT 10: *p* = 0.0595). However, MIT (5 and 10 mg/kg) significantly (*p* = 0.0001) decreased NMDAε2 expression compared to the control (Fig. 3).

### MIT decreases the expression of pCaMKIIα, pERK, pCREB, and BDNF

**Fig. 4:**
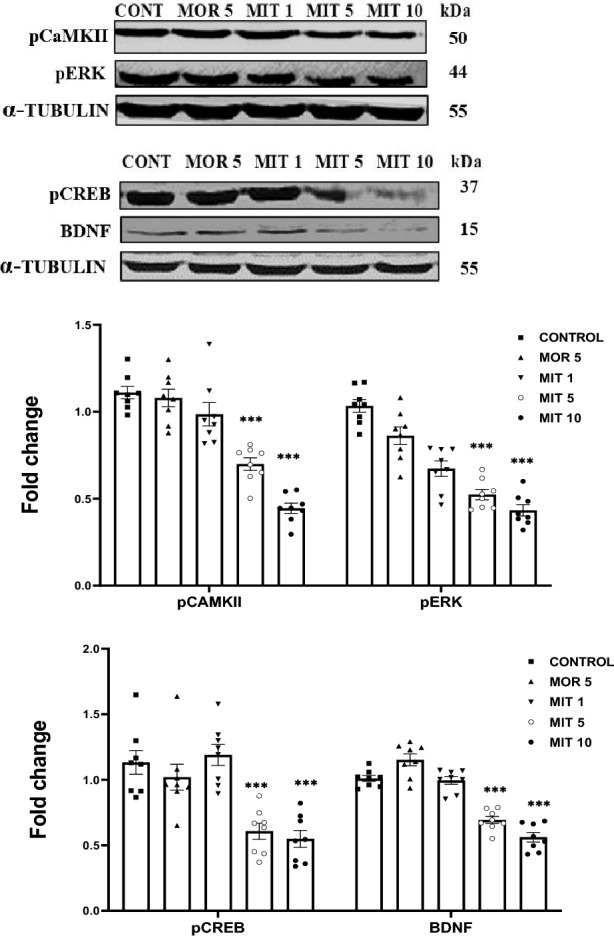
The effects of repeated administration of MOR (5 mg/kg) and MIT (1, 5, and 10 mg/kg) on pCaMKIIα, pERK, pCREB, and BDNF expression in the rats' hippocampus using western blot. Data are expressed as mean ± SEM and analyzed using one-way ANOVA followed by the Dunnett’s post hoc test (*n* = 8 replicates/group). **p < 0.005*,* ***p < 0.0001. Values in each group were calculated in relation to the control group and alpha-tubulin served as a loading control. *MOR* morphine, *MIT* mitragynine, *pCaMKIIα* phosphorylated calcium/calmodulin-dependent protein kinase type II alpha isoform, *pERK* phosphorylated extracellular regulated kinases, *pCREB* phosphorylated cAMP-response element binding protein, *BDNF* brain-derived neurotrophic factor

A one-way ANOVA revealed that expression of the phosphorylated CaMKIIα, ERK, CREB as well as expression of BDNF were all significantly downregulated (*p* < 0.005) by MIT (5 and 10 mg/kg) as compared to control in the hippocampus, whereas MIT (1 mg/kg) and MOR (5 mg/kg) did not yield significant changes the protein expression (Fig. 4).

### MIT reduces the expression of synaptophysin and PSD-95

**Fig. 5:**
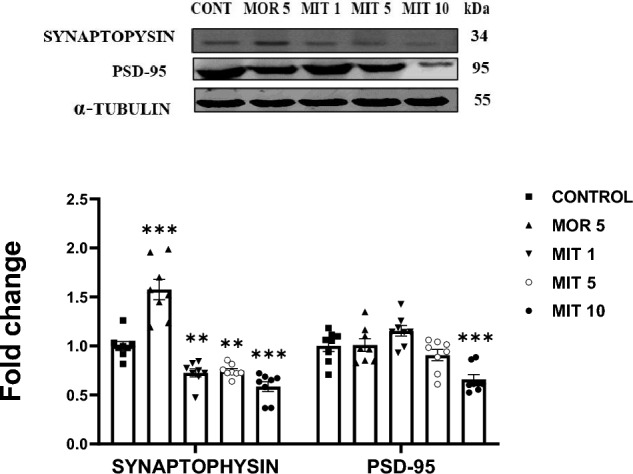
The effects of repeated administration of MOR (5 mg/kg) and MIT (1, 5, and 10 mg/kg) on synaptophysin and PSD-95 expression in the rats' hippocampus using western blot. Data are expressed as mean ± SEM and analyzed using one-way ANOVA followed by the Dunnett’s post hoc test (*n* = 8 replicates/group). **p < 0.001, ***p < 0.0001. Values in each group were calculated in relation to the control group and alpha-tubulin served as a loading control. MOR—morphine, MIT—mitragynine, PSD-95—post-synaptic density protein 95

A one-way ANOVA revealed that MIT at all the doses tested (1, 5, and 10 mg/kg) significantly decreased the expression of synaptophysin in the rat hippocampus (MIT 1: *p* = 0.0046*;* MIT 5*: p* = 0.0096*;* MIT 10: *p* < 0.0001). MOR 5 mg/kg produced a significant upregulation of synaptophysin (MOR 5: *p* < 0.0001) compared to the control. MIT (10 mg/kg) significantly decreased PSD-95 expression in the rats’ hippocampus as compared to the control (*p* = 0.0003), while MOR (5 mg/kg) and MIT (1 and 5 mg/kg) did not yield a significant difference in PSD-95 expression (MOR 5: *p* = 0.9998; MIT 1: *p* = 0.1944; MIT 5: *p* = 6119) Fig. 5.

### MIT reduces the expression Delta fosB and CDK-5

**Fig. 6:**
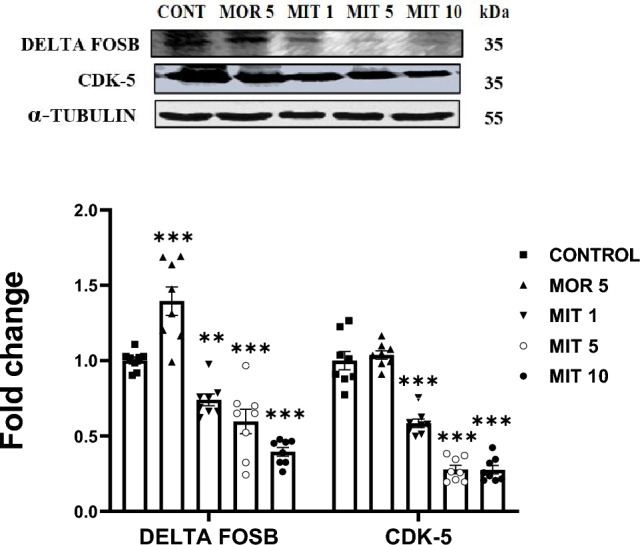
The effects of repeated administration of MOR (5 mg/kg) and MIT (1, 5, and 10 mg/kg)on delta FosB and CDK-5 expression in the rats' hippocampus using western blot. Data are expressed as mean ± SEM and analyzed using one-way ANOVA followed by the Dunnett’s post hoc test (*n* = 8 replicates/group). **p < 0.001, ***p < 0.0001. Values in each group were calculated in relation to the control group and alpha-tubulin served as a loading control. *MOR* morphine, *MIT* mitragynine, *CDK-5* cyclin-dependent kinase 5

A one-way ANOVA revealed that MIT (1, 5, and 10 mg/kg) significantly decreased Delta fosB (MIT 1: *p* = 0.0018; MIT 5: *p* < 0001; MIT 10: *p* < 0.0001) and CDK-5 expression (MIT 1:* p* < 0.0001; MIT 5: *p* < 0.0001; MIT 10: *p* < 0.0001) in the rat hippocampus when compared to control. However, expression of Delta fosB was significantly increased (*p* < 0.0001) in the hippocampus of MOR (5 mg/kg)-treated rats. Expression of CDK-5 remained unaffected in MOR (5 mg/kg) treated rats (*p* = 0.9557, Fig. 6).

## Discussion

Scientific evidence has shown that MIT causes cognitive impairments by affecting different types of learning and memory [10–12, 15]. LTP in the hippocampus is a crucial physiological mechanism underlying learning and memory processes [18]. Findings on synaptic plasticity have indicated that particular learning processes can expand the size of the dendritic spines, particularly at pyramidal neurons inside the CA1 arena of the hippocampus. This resulted in the development of new or strengthened synapses, which may act as the primary component of memory [47]. Recent studies demonstrated mild LTP suppression in the CA1 region of the hippocampus following acute treatment with MIT in rats [15]. In this work, we investigated the electrophysiological properties at the Schaffer collateral–CA1 synapses of 14-day MIT-treated rats in continuation of our previous study [15].

In the present study, MIT (5 and 10 mg/kg) significantly lowered the basal synaptic transmission in the hippocampus by decreasing the fEPSP amplitude at the stimulation intensities of 0.4–1.0 mA (Fig. 1a). We further assessed the effects of the treatments on both short- and long-term synaptic plasticity. PPF is a type of short-term synaptic plasticity induced by two stimulation pulses with a short interstimulus interval (ISI) that leads to calcium accumulation needed to cause neurotransmitter release from the pre-synaptic neuron [48, 49]. MIT at all the doses appeared not to have significant effects on the PPF ratio before TBS compared to control. However, MIT (10 mg/kg) significantly suppressed the PPF ratio after TBS at 100 and 200 interstimulus intervals suggesting a decrease in calcium influx necessary for short-term synaptic plasticity. The ability of MIT to suppress the PPF ratio at 100 and 200 interstimulus intervals suggests that these durations represent the optimum stimulation intervals needed for the calcium release. However in our earlier work [15], we reported that MIT does not affect short-term synaptic plasticity via PPF [15]. This may be because Ref. [15] reported the effect of acute exposure to MIT on PPF, while this study reported the effects of 14 days of repeated exposure to MIT on PPF.

LTP, a permanent synaptic process in the hippocampus, is regarded as the physiological basis for learning and memory [18, 50–53]. This study detected a significant and persistent LTP depression with MIT 5 mg/kg for up to 50 min duration by reducing the fEPSP amplitude. MIT (10 mg/kg) produced a profound LTP depression for almost 120 min, suggesting a marked interference with long-term memory. These findings support earlier reports on MIT, which, at higher doses, disrupts hippocampal synaptic transmission [15]. It also agrees with other research showing LTP suppression in rat hippocampus slices after administering kratom extracts [13, 54]. Interestingly, a lower dose of MIT (1 mg/kg) did not affect PPF or LTP, suggesting that this low dose is safe regarding synaptic transmission.

On the other hand, we found that, after MOR (5 mg/kg) administration, fEPSP amplitude remained unchanged in both PPF and LTP, thus suggesting morphine did not affect both short- and long-term synaptic plasticity in this study. This unique finding identified different actions between MOR and MIT. Although both MOR and MIT interact with opioid receptors to elicit anti-nociceptive action, the disruptive effects of MIT on learning processes do not seem to be mediated by opioid receptors. Many non-opioid mechanisms are included in the pharmacology of MIT compared to MOR [55]. Naltrexone that antagonized MOR action did not antagonize MIT effects which indicates a non-opioid mechanism exhibited by MIT [55]. MIT’s complex pharmacology is favored by its interactions with other receptors such as serotonergic [56], dopaminergic, cholinergic [57], and adrenergic receptors [58], as well as with many liver microsomal enzymes [59] leading the variety of pharmacological actions.

Neurotransmitters, particularly glutamate, are released during high-frequency stimulation of the CA3 region. The binding of glutamate to its receptors is a crucial step in the synaptic plasticity process [60]. Multiple exposures to psychostimulant substances have been shown to modify glutamatergic neurotransmission [61]. Glutamate overproduction results in synaptic dysregulation [62]. Our results indicate a significant extracellular glutamate level increase in the hippocampus of rats treated with MIT (5 and 10 mg/kg). Hippocampal glutamate levels in rats treated with MOR 5 mg/kg and MIT (1 mg/kg) were almost similar to those in vehicle-treated animals. Excessive glutamate neurotransmitter release is neurotoxic [63]. A glutamate accumulation in the hippocampus after repeated administration of MIT (5 and 10 mg/kg) may be responsible for neuronal death, which may constitute the reason behind the depressed basal synaptic transmission and LTP detected in this study. Because of the involvement of other brain regions in synaptic plasticity, we also tested the effects of our treatments on glutamate concentration in the midbrain. We observed a significant and dose-dependent increase in the glutamate level in all MIT-treated rats. However, the midbrain of rats treated with MOR (5 mg/kg) appears to have almost the same glutamate concentration as vehicle-treated rats, suggesting lower neurotoxicity of MOR compared to MIT.

To further understand the mechanism of neuroplasticity and memory following sub-chronic administration of MIT in rats, western blotting was employed to assess the changes in the expression of downstream proteins such as GluR-1, NMDAε2, pCaMKIIα, pERK, pCREB, BDNF, synaptophysin, PSD-95, Delta fosB, and CDK-5. These proteins have been associated with synaptic plasticity. All the treatment groups, including MOR (5 mg/kg), had no significant effect on GluR-1 expression. However, MIT (5 and 10 mg/kg) significantly decreased NMDAε2 expression. Dysregulations of the AMPA and NMDA receptors have been linked to changes in synaptic transmission in the brain [64, 65].

Glutamatergic neurotransmission via NMDA-R activates new protein formation and induces neuroprotection via activation of CaMKIIα, ERK, CREB, and BDNF signaling pathways which are the major proteins associated with LTP [66]. Alterations in the ERK/CREB/BDNF pathway were reported to deplete synaptic plasticity and memory [67]. We, therefore, asked whether the modulation of hippocampal synaptic transmission observed in this study could be due to the changes in these proteins' expression. According to Ref. [68], CaMKII, a highly expressed protein in post-synaptic neurons of the hippocampus, has remained a crucial molecule linked to synaptic plasticity. It is activated by interactions with Ca^2+^ and other synaptic proteins like the NMDA-R, resulting in phosphorylation and increased channel conductance essential for synaptic plasticity [69, 70]. We observed a significant decrease in the phosphorylated form of CaMKIIα (pCaMKIIα) expression after MIT (5 and 10 mg/kg). pCaMKIIα is the activated type of CaMKIIα. This data might imply that repeated MIT exposure decreases CaMKIIα phosphorylation via NMDAε2 dysregulation, thereby causing LTP decline. The repeated exposure to 1 mg/kg MIT and 5 mg/kg MOR did not impact CaMKIIα phosphorylation. Numerous findings have demonstrated the significance of NMDA-R-dependent activation of CaMKII in the molecular process of LTP and learning [22, 23, 62–65].

Another signaling crucial to synaptic plasticity and memory is the ERK/CREB pathway which is activated following the induction of LTP and behavioral training [71]. ERK is a densely expressed protein kinase that has been implicated as playing a critical role in neuroplasticity (Um et al., 2018). Its inhibition leads to a decline in LTP [72]. ERK activation regulates CREB phosphorylation, which has been reported to occur during LTP induction [72, 73]. The late phase of LTP has been demonstrated to be critical for ERK signaling. Synaptic potentiation also activates Ca^2+^ and NMDA-R-dependent ERK signaling [68]. In rats, hippocampal LTP was impaired following the inhibition of the CaMKII/ERK/CREB signaling pathway [21]. In this study, MIT 5 and 10 mg/kg appeared to disrupt ERK and CREB phosphorylation significantly. The transcription factor CREB is activated by ERK signaling to promote gene expression [69, 70]. Expression of other proteins related to neuroplasticity, such as BDNF, was reported to be altered in response to CREB modulation [74]. Furthermore, it has been shown that BDNF has lasting impacts on maintaining LTP, primarily due to its role in promoting the biosynthesis of proteins and structural modification at the synaptic level [74, 75]. LTP is facilitated at Schaffer collateral–CA1 synapses when BDNF interacts with TrkB (tropomyosin-like receptor kinase B), which serves as its receptor [74]. MIT 5 and 10 mg/kg significantly decreased the expression of BDNF in our current study.

Decreased expression of pCaMKIIα, pERK, pCREB, and BDNF in the rat hippocampus was reported to impair LTP and learning [75, 76], and other forms of synaptic plasticity [77]. The observed activity-dependent modulation of these synaptic plasticity proteins may constitute a molecular pathway of how MIT induces suppression of hippocampal plasticity and subsequent cognitive deficits.

Alterations of the synaptic structures and density in the hippocampus are considerably associated with synaptophysin and PSD-95 expression [78] and consequently affect synaptic plasticity and learning [79]. Synaptophysin is considered a reliable indicator for determining the distribution and density of synapses [80, 81]. In this study, MIT at all the doses tested significantly decreased synaptophysin expression dose dependently. These data suggest that MIT administration for 14 days disrupts synaptogenesis, affecting synaptic plasticity. Interestingly, MOR (5 mg/kg) significantly increased synaptophysin expression as compared to control, and thus may enhance synaptogenesis. These findings align with previous reports of increased structural plasticity after MOR treatment and self-administration in rats [81, 82]. This finding may also be one of the reasons why MOR (5 mg/kg) did not alter LTP in this study. Although MOR and MIT interact with opioid receptors and elicit analgesic effects, MOR was reported to block LTP completely because it decreased GABAergic neurotransmission via a guanylate cyclase interaction [79].

PSD 95 is essential for regulating NMDA-R gating, trafficking, and intracellular signaling in response to synaptic plasticity by directly binding to NMDA-Rs [81], thus allowing an influx of Ca^2+^ necessary during LTP induction. Here, we recorded a profound downregulation of PSD 95 after MIT (10 mg/kg) treatment. This result indicates that MIT affects NMDAε2 conductance via PSD 95 dysregulation. The latter could cause the MIT-inhibited Ca^2+^ influx that was previously observed [15].

Delta fosB is a C-terminal truncated FosB gene product generated by alternative splicing. It is a molecular mediator of long-term plasticity in the brain [83]. Delta fosB gene expression depends on Ca^2+^ influx and subsequent activation of the mitogen-activated protein kinase ERK1/2 [36], a prominent protein implicated in neuroplasticity. Our data indicate that MIT decreased the expression of Delta fosB. It may be the reason behind the ERK/CREB signaling pathway disruption observed in this study. MOR 5 mg/kg produced an opposite effect to MIT by significantly upregulating the expression of Delta fosB.

CDK-5 is an essential protein for synaptic modulation on both pre- and post-synaptic neurons. It has been linked to alterations in synaptic strength and connectivity of hippocampal CA3 recurrent synapses [37]. The development and retraction of dendritic spines and the conductance and expression of NMDA-Rs are regulated by CDK-5 phosphorylation [84]. CDK-5 also causes an increase in pre-synaptic N-type voltage-gated calcium channel opening probability [85], which plays a role in neurotransmitter release and neuroplasticity. This work detected a significant decrease in CDK-5 expression after all MIT treatments.

Altogether, present findings suggest that repeated exposure to MIT at a lower dose (1 mg/kg) in rats is safe; however, higher doses (5 and 10 mg/kg) disrupt hippocampal synaptic transmission dose dependently via NMDAε2, CaMKII, ERK, CREB, BDNF, synaptophysin, PSD-95, Delta fosB, and CDK-5 modifications. It may represent the molecular mechanism of how MIT induces LTP deficit and subsequent cognitive impairments, and this may serve as a potential therapeutic target to manage MIT effects on LTP. In addition, we found that MIT behaves differently from MOR in terms of synaptic plasticity.

### Supplementary Information

Below is the link to the electronic supplementary material.

Supplementary file1 (DOCX 1877 KB)

## Data Availability

The datasets generated during and/or analyzed during the current study are available from the corresponding author upon reasonable request.

## Abbreviations

AMPA-R: Alpha-amino-3-hydroxy-5-methyl-4-isoxazole propionic acid receptor
ANOVA: Analysis of variance
ARASC: Animal Research and Service Centre
BDNF: Brain-derived neurotrophic factor
CaMKII: Calcium/calmodulin-dependent protein kinase II
CDR: Centre for drug research
CA1: Cornu ammonis 1
CA3: Cornu ammonis 3
CREB: Cyclic AMP-response element binding protein
DG: Dentate gyrus
DTT: Dithiothreitol
GLU: Glutamate
ERK: Extracellular signal-regulated kinase
fEPSP: Field excitatory post-synaptic potentials
HP: Hippocampus
I/O: Input–output
IACUC: Institutional Animal Care and Use Committee
ISIs: Interstimulus intervals
LTP: Long-term potentiation
MB: Midbrain
MIT: Mitragynine
MOR: Morphine
NMDA-R: N-methyl-D-aspartic acid receptor
PPF: Paired-pulse facilitation
PSD-95: Post-synaptic density protein 95
SDS: Sodium dodecyl sulfate
SD: Sprague Dawley
SEM: Standard error of the mean
TBS: Theta-burst stimulation
TBS-T: Tris-buffered saline/Tween 20
