# Supplementary material for: Mitragynine inhibits hippocampus neuroplasticity and its molecular mechanism
Source: Pharmacol Rep. 2023 Nov 4;75(6):1488–501. doi: 10.1007/s43440-023-00541-w (PMC10661785; doi:10.1007/s43440-023-00541-w)
Supplement: Supplementary file 1 — Supplementary file1 (DOCX 1877 KB) [file 43440_2023_541_MOESM1_ESM.docx]

Supplementary figures


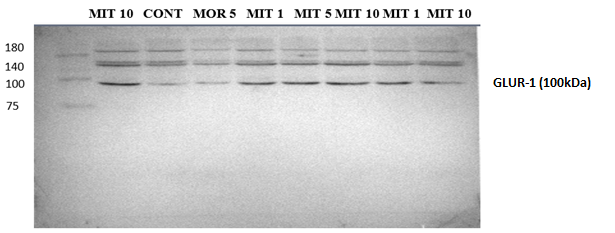


**Fig. S1:**  Glutamate receptor 1 (GluR-1) expression in the hippocampus of rats after repeated administration of morphine (MOR, 5 mg/kg) or mitragynine (MIT, 1, 5, or 10 mg/kg). Shown are exemplary immunoblots.


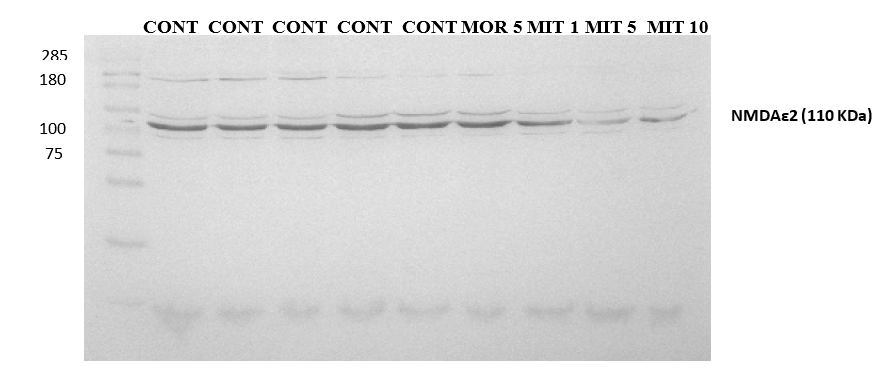


**Fig. S2:**  NMDA epsilon 2 (NMDAε2) expression in the hippocampus of rats after repeated administration of morphine (MOR, 5 mg/kg) or mitragynine (MIT, 1, 5, or 10 mg/kg). Shown are exemplary immunoblots.


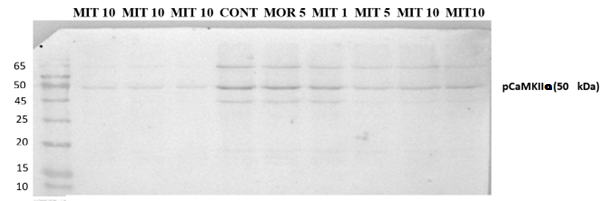


**Fig. S3:** Phosphorylated calcium/calmodulin-dependent protein kinase type II alpha isoform

(pCaMKIIα) expression in the hippocampus of rats after repeated administration of morphine (MOR, 5 mg/kg) or mitragynine (MIT, 1, 5, or 10 mg/kg). Shown are exemplary immunoblots.


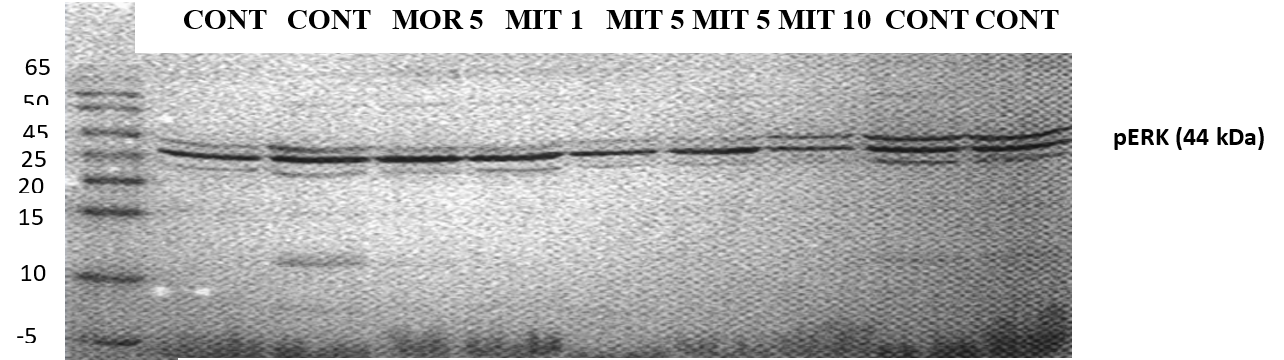


**Fig. S4:** Phosphorylated extracellular regulated kinases (pERK) expression in the hippocampus of rats after repeated administration of morphine (MOR, 5 mg/kg) or mitragynine (MIT, 1, 5, or 10 mg/kg). Shown are exemplary immunoblots.


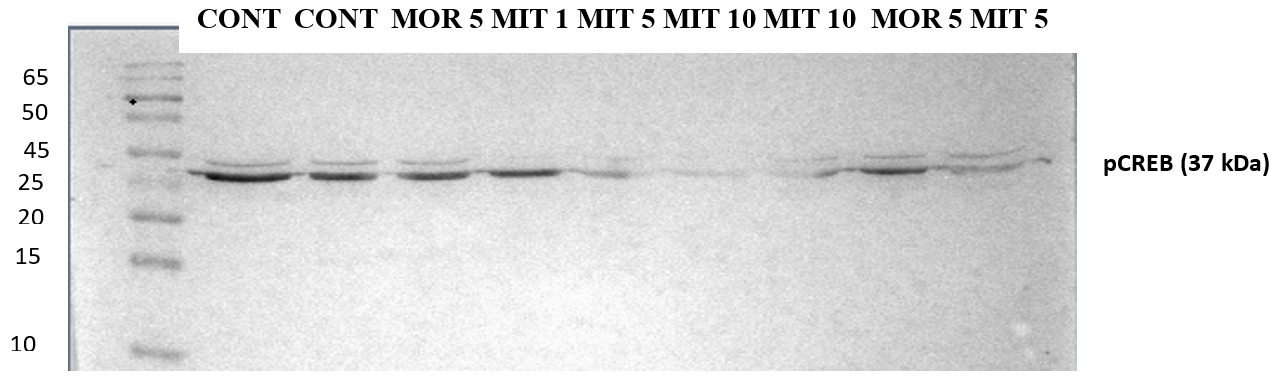


**Fig. S5:** Phosphorylated cAMP response element-binding (pCREB) expression in the hippocampus of rats after repeated administration of morphine (MOR, 5 mg/kg) or mitragynine (MIT, 1, 5, or 10 mg/kg). Shown are exemplary immunoblots.


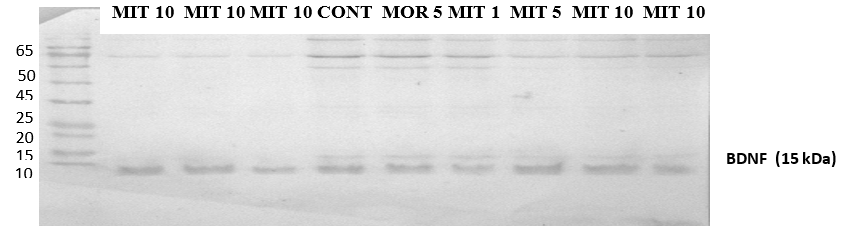


**Fig. S6:** Brain-derived neurotrophic factor (BDNF) expression in the hippocampus of rats after repeated administration of morphine (MOR, 5 mg/kg) or mitragynine (MIT, 1, 5, or 10 mg/kg). Shown are exemplary immunoblots.


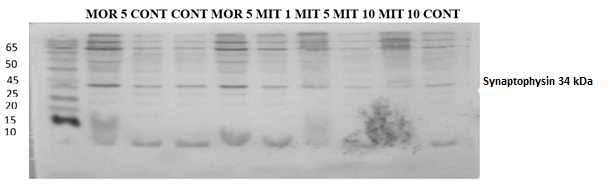


**Fig. S7:** Synaptophysin expression in the hippocampus of rats after repeated administration of morphine (MOR, 5 mg/kg) or mitragynine (MIT, 1, 5, or 10 mg/kg). Shown are exemplary immunoblots.


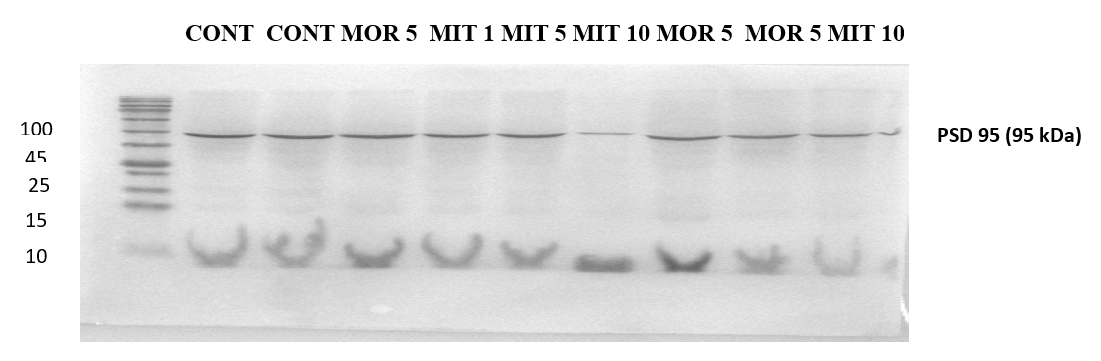


**Fig. S8:** Postsynaptic density protein 95 (PSD 95) expression in the hippocampus of rats after repeated administration of morphine (MOR, 5 mg/kg) or mitragynine (MIT, 1, 5, or 10 mg/kg). Shown are exemplary immunoblots.


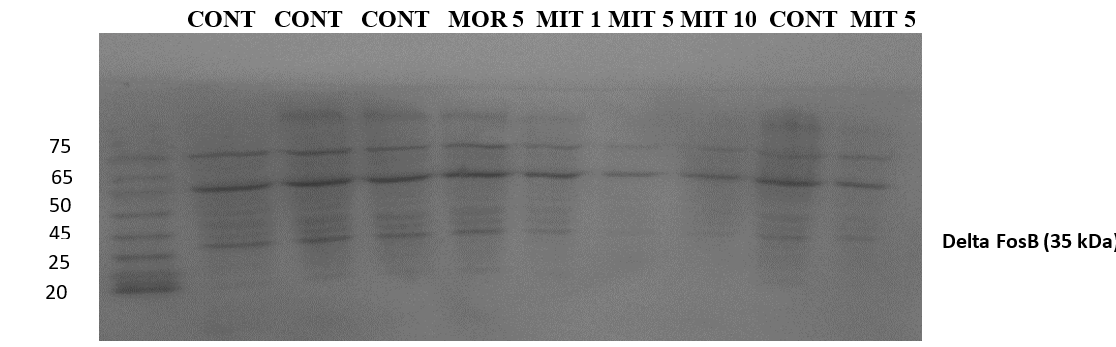


**Fig. S9:** Delta FosB expression in the hippocampus of rats after repeated administration of morphine (MOR, 5 mg/kg) or mitragynine (MIT, 1, 5, or 10 mg/kg). Shown are exemplary immunoblots.


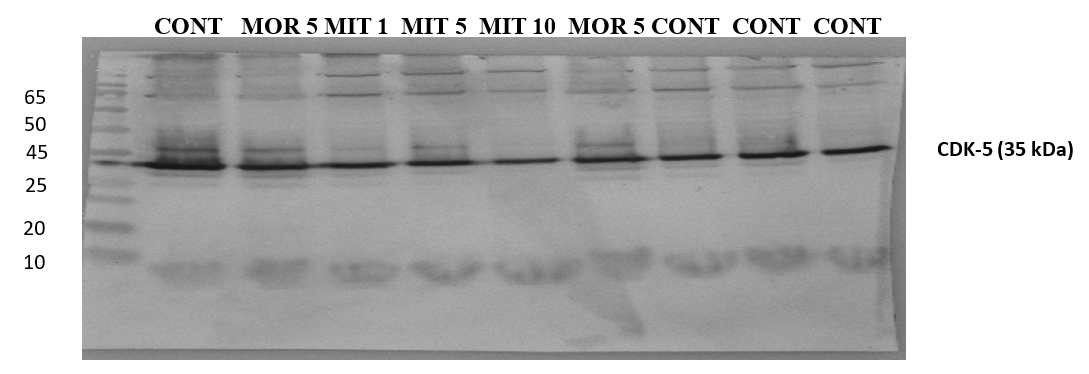


**Fig. S10:** Cyclin-dependent kinase 5 (CDK-5) expression in the hippocampus of rats after repeated administration of morphine (MOR, 5 mg/kg) or mitragynine (MIT, 1, 5, or 10 mg/kg). Shown are exemplary immunoblots.


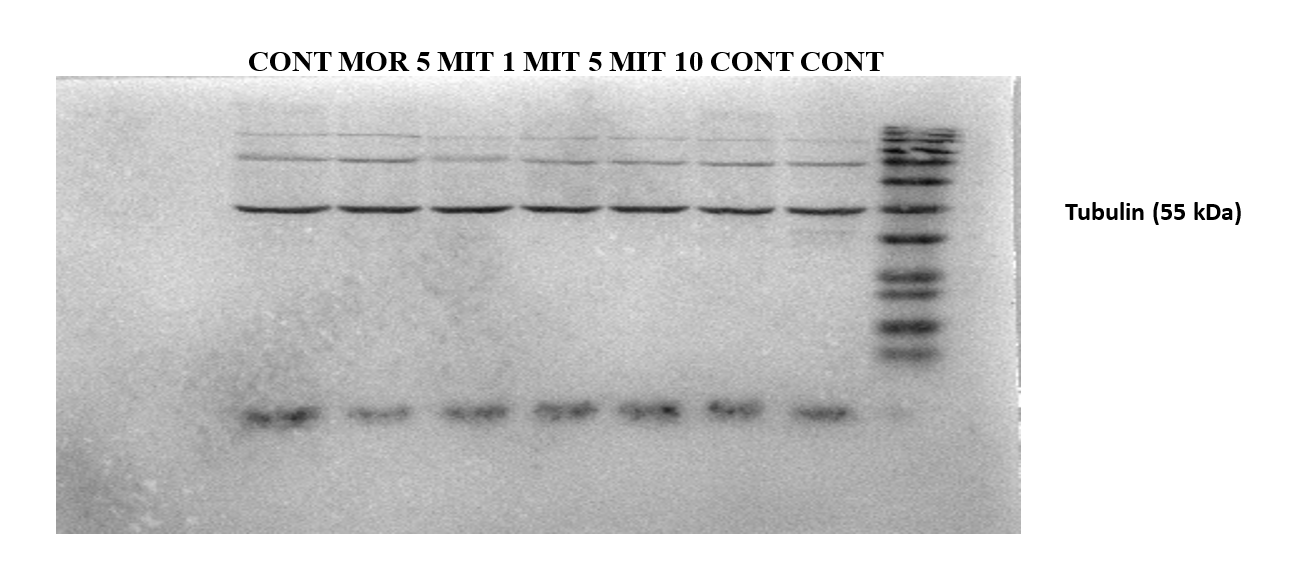


**Fig. S11:** Alpha tubulin expression in the hippocampus of rats after repeated administration of morphine (MOR, 5 mg/kg) or mitragynine (MIT, 1, 5, or 10 mg/kg). Shown are exemplary immunoblots.
